# Using part of the initial analgesic dose as the epidural test dose did not delay the onset of labor analgesia: a randomized controlled clinical trial

**DOI:** 10.1186/s12884-024-06475-2

**Published:** 2024-04-08

**Authors:** Jianxiao Chen, Sumeng Chen, Hao Lv, Peijun Lv, Xinhua Yu, Shaoqiang Huang

**Affiliations:** 1 Department of Anesthesia, Shaoxing Shangyu Maternal and Child Health Hospital, 35 Banshan Road, Shangyu Block, Shaoxing, Zhejiang China; 2 Department of Anesthesia, Obstetrics & Gynecology Hospital of Fudan University, 128 Shenyang Road, Yangpu Block, Shanghai, China; 3 Division of Epidemiology, Biostatistics and Environmental Health, School of Health, University of Memphis, 3770 Desoto, Memphis, USA

**Keywords:** Labor analgesia, Test dose, Epidural anesthesia, Ropivacaine, Lidocaine

## Abstract

**Background:**

Epidural test dose for labor analgesia is controversial and varies widely in clinical practice. It is currently unclear whether using a portion of the initial dose for analgesia as the test dose delays the onset time of analgesia, compared to the traditional test dose.

**Methods:**

One hundred and twenty-six parturients who chose epidural analgesia during labor were randomly assigned to two groups. The first dose in group L was 3 ml 1.5% lidocaine, and in the RF group was 10 ml 0.1% ropivacaine combined with 2 μg/ml fentanyl. After 3 min of observation, both groups received 8 ml 0.1% ropivacaine combined with 2 μg/ml fentanyl. The onset time of analgesia, motor and sensory blockade level, numerical pain rating scale, patient satisfaction score, and side effects were recorded.

**Results:**

The onset time of analgesia in group RF was similar to that in group L (group RF vs group L, 7.0 [5.0–9.0] minutes vs 8.0 [5.0–11.0] minutes, *p* = 0.197). The incidence of foot numbness (group RF vs group L, 34.9% vs 57.1%, *p* = 0.020) and foot warming (group RF vs group L, 15.9% vs 47.6%, *p* < 0.001) in group RF was significantly lower than that in group L. There was no difference between the two groups on other outcomes.

**Conclusions:**

Compared with 1.5% lidocaine 3 ml, 0.1% ropivacaine 10 ml combined with 2 μg/ml fentanyl as an epidural test dose did not delay the onset of labor analgesia, and the side effects were slightly reduced.

**Clinical trial registration:**

http://www.chictr.org.cn (ChiCTR2100043071).

## Introduction

Epidural analgesia is the most effective and widely used method to alleviate delivery pain in clinical practice [1, 2]. Unexpected insertion of the epidural catheter into vessels or subarachnoid space may lead to local anesthetics systemic toxicity (LAST) or total spinal anesthesia, which are life-threatening [3].

Therefore, a test dose with low concentration and volume of local anesthetics, is usually injected to ensure that the cannula is in the epidural space before a larger dose of local anesthetics infused through the catheter [4, 5]. Classical test doses include 45 mg lidocaine and 15 μg epinephrine [6]. However, “standard” test doses in obstetrics have been reported to be associated with a range of serious adverse events and deaths [7]. Meanwhile, the magnitude of changes in maternal heart rate is significantly increased due to contractions after labor, which may mask the expected hemodynamic changes from epinephrine. Considering the limited maternal sensitivity of epinephrine testing and potential adverse effects such as increased myocardial oxygen consumption and decreased uteroplacental blood flow, the addition of epinephrine to the epidural test dose is considered to do more harm than good [8].

The concentration of local anesthetics used in labor analgesia is low (ropivacaine is usually no more than 0.15%), and the volume of first doses is no more than 10 ml [9–12]. With such low concentration and volume of local anesthetics, the risks of LAST and total spinal anesthesia are little even when local anesthetics are injected into the circulation or subarachnoid space unexpectedly.

Therefore, many hospitals use a portion of the initial analgesic dose as a test dose in clinical practice [13]. Our previous study in maternal has also shown that ropivacaine 5 mg in combination with a low-dose opioid is effective and safe as an epidural test dose to detect intrathecal injection [14]. For the maternal with cardiac disease, a scientific statement from the American Heart Association (AHA) also recommends identifying intravascular and intrathecal administration by low-dose fentanyl and local anesthetics at analgesic concentrations, respectively [15]. However, the onset time of lidocaine (5–15 min) is shorter than that of ropivacaine (15–20 min) in epidural anesthesia [16]. It is still not clear whether the onset time of epidural analgesia is prolonged or not if a low dose of ropivacaine is used as a test dose instead of lidocaine. Although it is convenient to use part of the analgesic solution as a test dose, waiting too long for pain relief can also be a disadvantage for a woman experiencing painful contractions. The purpose of this prospective randomized controlled trial is to compare the characteristics of epidural labor analgesia induced by the traditional test dose with the use of a portion of the initial analgesic dose as a test dose. The primary outcome is the onset time of analgesia.

## Material and methods

### Study design and participants

This study was approved by the Ethics Committee of Shangyu Maternal and Child Health Care Hospital of Zhejiang Province and has been registered on 04/02/2021 in the Chinese Clinical Trial Registry (http://www.chictr.org.cn) as ChiCTR2100043071. This article adhered to the applicable Consolidated Standards of Reporting Trials (CONSORT, Author Checklist) guidelines [17]. All participants are voluntary and have signed informed consent.

This study involved 140 parturient women who requested labor analgesia during vaginal delivery in Shangyu Maternal and Child Health Care Hospital between February to July 2021. The inclusion criteria are American Society of Anesthesiologists (ASA) grade I-II; Singleton primipara aged 18–40 years old, pre-assessment can be vaginal delivery; Height 155–170 cm, BMI 18.5–35 kg/m^2^; Cervical dilation 2–6 cm; No obvious history of cardiopulmonary disease and no history of surgical trauma; Under the provisions of the Good Clinical Practice (GCP), obtain informed consent and volunteer. The exclusion criteria include contraindications of neuraxial block, physical or mental disabilities, such as scoliosis helical and depression, alcohol or drug abuse, diseases of essential organs, such as hyperthyroidism, cardiopulmonary disease, diabetes treated with insulin and neuromuscular diseases, numeric pain rating scale (NPRS) < 5, and refusion to attend the study. Included participants would be excluded if the following situations occur: failure of epidural block (failure of epidural catheter placement or failure to be effective after drug injection), accidental dural puncture, blood in the epidural catheter after negative aspiration, failure to follow medication protocols, deciding to have a cesarean section in 30 min after epidural analgesia and missing data records after obtaining informed consent.

### Randomization and blinding

Anesthesiology nurses who are not involved in observation and evaluation were used the randomized website (https://www.randomizer.org) to generate a random number table and randomly assign all participants to group RF (0.1% ropivacaine and 2 μg/ml fentanyl in 10 ml) or group L (1.5% lidocaine in 3 ml). The anesthesiologist will be known of the grouping information due to the different volumes of the test dose. To be as blind as possible, both the assessor and the parturient were blinded to the grouping.

### Study procedures

**Fig. 1:**
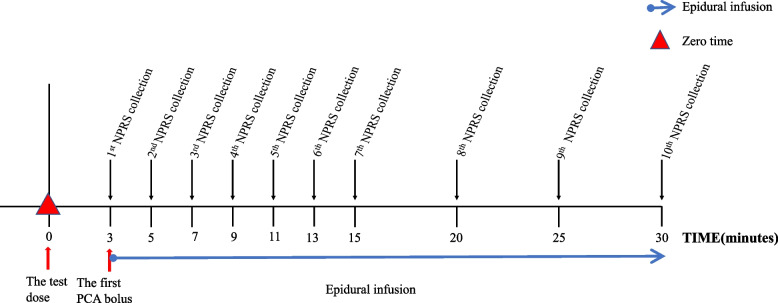
Flowchart of epidural dosing and pain score assessment

After the intravenous cannula was inserted, the mother lied on the left lateral position. Experienced anesthesiologists started disinfection and epidural puncture between L2-L3 or L3-L4. After the resistance-to-saline loss, the epidural catheter was inserted 3–4 cm in depth towards the cephalad direction and secured with sterile dressing and tape. As soon as the epidural catheter was secured, pregnant women returned to the supine position of the left leaning uterus. Modified test dose, 0.1% ropivacaine (AstraZeneca AB, Sweden) and 2 μg/ml fentanyl (Yichang Humanwell Pharmaceutical Co., Ltd., Hubei, China) in 10 ml, or traditional test dose, 1.5% lidocaine (Shanghai Harvest Pharmaceutical Co., Ltd., China) in 3 ml were injected into the participant through the epidural catheter in one shot. After 3 minutes of close monitoring to prevent LAST and spinal anesthesia, participants were connected to an epidural analgesia pump. The content of the analgesic pump in both groups was 0.1% ropivacaine combined with 2 μg/ml fentanyl. The background infusion rate was 8 ml/h, patient-controlled-epidural-analgesia (PCEA) bolus was 8 ml, and locked time was 20 min. The first PCEA dose was given as soon as the analgesic pump was connected. Then parturient women were closely observed for 30 min. The timing of epidural dose injection and data collection is detailed in Fig. 1. All procedures were performed by anesthesiologists with more than 10 years of experience. Observation outcomes, including the onset of analgesia, NPRS, sensory and motor blocks and so on, were assessed and recorded on standard forms by a trained anesthetic nurse.

### Outcome measures

The zero time point (0 min) is defined as immediately after the injection of the first dose, the epidural test dose. NPRS (0 means no pain and 10 means unendurable pain) was evaluated every 2 min from 3 min to 15 min and every 5 min from 15 min to 30 min. The sensory block level was measured by pin-prick testing on the medioventral line. A modified Bromage scale was used to evaluate motor blocking (0: no motor block; 1: can’t lift legs; 2: can’t bend knees; 3: can’t bend malleolus). The sensory and motor blocking were evaluated every 2 min from 3 min to 15 min and every 5 min from 15 min to 30 min after the test dose was given. BP, HR, and SpO_2_ were monitored every 5 min from 0 min to 30 min. Hypotension was defined as systolic blood pressure (SBP) < 90 mmHg or a decrease of SBP > 20% baseline, and hypoxemia was defined as SPO_2_ < 92%. Once hypotension occurred, the parturient was turned to the left lateral position, the infusion was accelerated, and 50 μg phenylephrine was given if needed. Other complications, like foot warming, foot numbness, shivering, pruritus, headache, tinnitus of the parturient, or fetal intrauterine distress, would also be recorded during the labor analgesia. At 30 min after epidural administration of the test dose, patient satisfaction scales were conducted (0, completely dissatisfied; 1, neutral; 2, satisfied; and 3, completely satisfied).

The onset time of analgesia was defined as NPRS ≤3 or the reduction of NPRS ≥50% of the baseline value. If the analgesic onset failed at 30 min, 10 ml of 0.1% ropivacaine and 2 μg/ml fentanyl were given again. If the additional dose lowered NPRS and enabled analgesic onset of the parturient, the data of such participants would be included in this study, otherwise it would be considered a failure of epidural analgesia, and accordingly the mothers were excluded from the statistical analysis.

The primary outcome of this study was the onset time of analgesia. And the secondary outcomes included patient NPRS at different time point, the highest sensory block level, the maximum motor block level, the incidence of foot numbness and foot warming, the patient satisfaction scores and the incidence of hypotension.

### Statistical analysis

In our pilot study, the mean onset time of analgesic induction regimen containing traditional test dose was 8 minutes and the standard deviation was 3.92 minutes. The difference of analgesic onset time between the two groups that are thought to be of clinical significance is *≥*2 min [18]. At a two-side type I error α value of 0.05, and type II error β of 0.20 (power of 80%), the sample size for each group was calculated to be at least 62. Considering a 10% drop out ratio, a final sample of 70 participants for each group were recruited.

The normally distributed data were shown as Mean (SD), while the abnormally distributed data were shown as Median (Interquartile range [range]). The data that had normal distribution and variance homogeneity was analyzed by the independent sample t-test. While the data that didn’t meet these two conditions at the same time were analyzed by the Mann-Whitney U test. Categorical variables were analyzed using the Chi-square test or Fisher exact test. The onset time of labor analgesia in the two groups were shown as Kaplan-Meier survival curve. Hazard ratios (HRs) for the two groups were using the Cox Proportional Hazard Regression model. Statistical analysis was performed using SPSS 26.0 (SPSS, Inc., Chicago, IL, USA). *P* value< 0.05 was considered statistically significant.

## Results

**Fig. 2:**
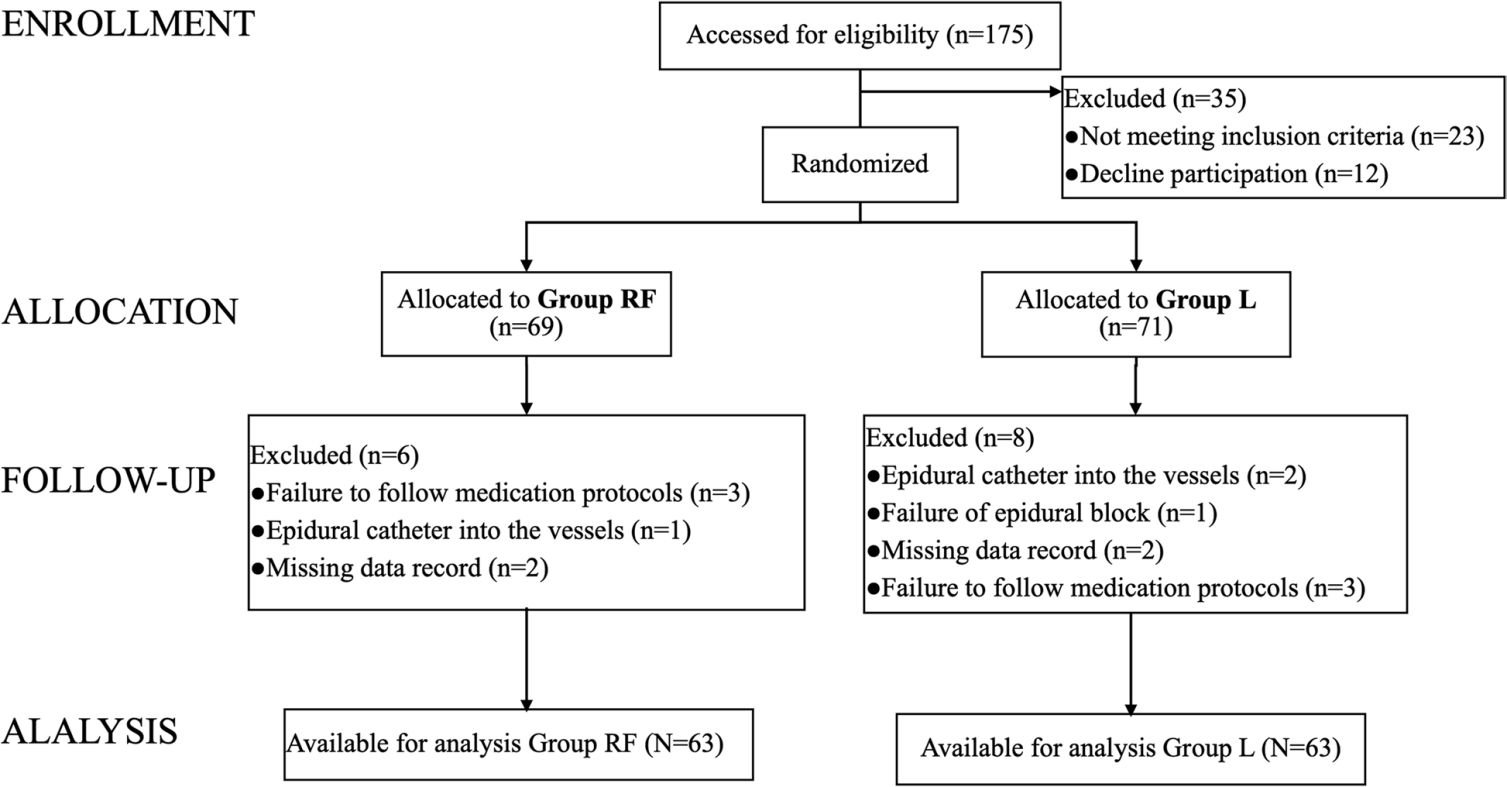
Consort recruitment diagram. Group RF: 0.1% ropivacaine and 2 μg/ml fentanyl; Group L: 1.5% lidocaine

A total of 140 parturients were assigned randomly to group L or group RF. Fourteen participants were excluded because of failure to follow medication protocols, epidural catheter into the vessels, missing data record or failure of epidural block. Finally, 126 participants (63 parturient in each group) were included in this study (Fig. 2). The baseline characteristics, including the age, weight, height, body mass index (BMI), initial NPRS, and oxytocin exposure of maternal women, are presented in Table 1. There was no difference between the two groups.

**Table 1:** The baseline characteristics of participants

	Group RF (*n* = 63)	Group L (*n* = 63)	*P*-value
Age (years)	27.0 [25.0–29.0]	27.0 [25.0–30.0]	0.253
BMI (kg/m^2^)	26.8 [24.1–29.1]	26.6 [24.2–29.1]	0.830
Weight (kg)	68.0(9.2)	69.4(9.5)	0.951
Height (cm)	160.0 [156.0–162.0]	160.0 [157.0–164.0]	0.173
Initial NPRS	7.5 [7.0–10.0]	7.0 [7.0–9.0]	0.604
Oxytocin0/1/2(U)	50/13/0	53/8/1	0.515

Data are expressed as mean (standard deviation) or median [interquartile range). Group RF: 0.1% ropivacaine and 2 μg/ml fentanyl; Group L: 1.5% lidocaine; BMI: body mass index = weight/height^2^

**Fig. 3:**
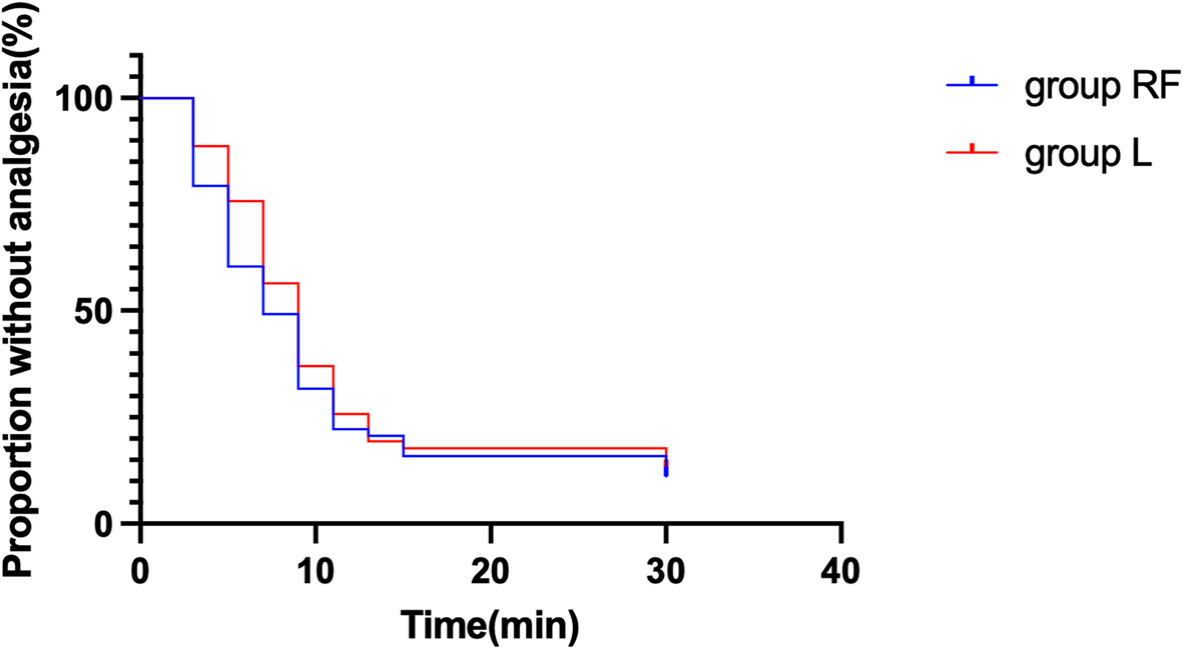
Kaplan-Meier survival curve for the onset time of analgesia in RF and L groups. The results of the Cox model (concomitant variables: age, height, weight) indicated that no difference between group RF and group L (*P* = 0.47)

The onset time of analgesia in group RF was similar to that in group L (group RF vs group L, 7.0 [5.0–9.0] minutes vs 8.0 [5.0–11.0] minutes, *p* = 0.197). We considered 30 min after the test dose was injected as the end point of observation and made Kaplan-Meier survival curves to show the onset time of analgesia in RF and L groups (Fig. 3). The results of the Cox model (concomitant variables: age, height, weight) for group RF versus group L were as follow: Hazard Ratio (HR)1.148, 95% CI 0.790–1.669, *p* = 0.47. These data showed no difference between the RF and L group.

There was no difference between the two groups regarding the time for NPRS to decrease by at least 2 points, the value of the lowest NPRS within 30 min, failed to reach the onset of analgesia, the highest sensory block level, the maximum motor block level, the patient satisfaction scores or the occurrence of hypotension (Table 2). However, the incidence of foot warming (group RF vs group L, 15.9% vs 47.6%, *p* < 0.001,) and foot numbness (group RF vs group L, 34.9% vs 57.1%, *p* = 0.020,) in group RF were significantly lower than those of group L.

**Table 2:** Summary of outcomes of participants

	Group RF (*n* = 63)	Group L (*n* = 63)	*P*-value
Onset of analgesia (min)	7.0 [5.0–9.0]	8.0 [5.0–11.0]	0.197
Failed to reach onset of analgesia	7 [11.1%]	9 (14.3%)	0.790
Time for NPRS to decrease ≥2 (min)	5.0 [3.0–7.0]	6.5 [3.0–9.0]	0.056
Lowest NPRS within 30 min	1.0 [0.0–2.5]	1.0 [0.0–3.0]	0.747
Highest sensory block level	T6 [T5-T8]	T6 [T6-T8]	0.212
Maximum motor block0/1/2/3	63/0/0/0	63/0/0/0	1.000
Satisfaction0/1/2/3 at 30 min	0/0/7/56	2/4/4/53	0.535
Foot warming	10 (15.9%)	30(47.6%)	0.000
Foot numbness	22 (34.9%)	36(57.1%)	0.020
Hypotension	1 (1.6%)	5(7.9%)	0.207

Values are shown as median [interquartile range] or number (proportion). Group RF: 0.1% ropivacaine and 2 μg/ml fentanyl; Group L: 1.5% lidocaine; NPRS: numeric pain rating scale, 0 means no pain and 10 means unendurable pain

## Discussion

This study compared the characteristics and side effects of analgesia initiated with a portion of the analgesic dose as the test dose and with a conventional epidural test dose during labor analgesia induction. The results showed that when 0.1% ropivacaine 10 ml combined with 2 μg/ml fentanyl was used as the epidural test dose to start labor analgesia, the onset time of analgesia was similar to that initiated by 1.5% lidocaine 3 ml, but the side effects of foot numbness and warmth were slightly reduced.

Tahir and his colleagues [4] first raised the concept of test dose during epidural anesthesia in obstetric patients in 1975. If the epidural catheter is inserted into vessels or subarachnoid space by mistake, a large dose of high-concentration local anesthetics would be injected into the blood circulation or subarachnoid space, leading to severe adverse effects. Thus, it is necessary to inject a test dose of 45 mg lidocaine and 15 μg epinephrine to ensure the right position of the epidural cannula during epidural anesthesia [4, 6]. However, more and more experts have doubted the safety and efficacy of traditional test doses in obstetric patients recently [7, 19]. Epinephrine administration to the parturient is the most criticized. Sometimes, it is difficult to identify whether the elevated HR is caused by epinephrine or by uterine contractions and labor pain, thus it is hard to determine whether local anesthetics are injected into the vessel or not through test dose [13]. Epinephrine may increase motor nerve block and slow down the labor stage. Although epinephrine administration in the epidural space has very limited systemic hemodynamic changes, it may increase myocardial oxygen consumption, worsen the hemodynamic situations of preeclampsia parturient, decrease the blood flow of uterine and placenta, cause fetal distress in the uterus and other adverse effects [8]. All these clinical evidences prove that the disadvantage of using epinephrine in epidural labor analgesia overweighs its advantage. Therefore, we did not use epinephrine even in the traditional test dose group. Opioids, which produce dizziness and drowsiness soon after being injected into the vein, can replace epinephrine to determine whether the catheter has entered the blood vessel [20]. Especially for the maternal with cardiovascular disease, a scientific statement from the AHA also recommends the use of low-dose fentanyl to identify if the epidural catheter is misplaced into the vessel [15]. Another method to determine whether an epidural catheter has entered the vascular system is to observe the epidural analgesic effect. Accidental intravenous cannulation presents with epidural failure, which requires a reassessment of the position of the epidural catheter and, if necessary, reinserts the catheter before systemic toxicity of the local anesthetic occurs [7].

In addition, the traditional test dose of lidocaine 45 mg is too high for parturient women. Several studies reported extensive sensory and motor block as well as severe hypotension after unexpected spinal block by epidural test dose, resulting in unplanned airway management and emergency cesarean section being related to fetal distress [21–23]. Some institutions reduce the dose of lidocaine to 30 mg, but Pratt et al. [24] did not identify a decrease in the rate of side effects with the lower dose in case of intrathecal injection. Epidural labor analgesia is routinely administered with low-dose-low-concentration local anesthetics, usually bupivacaine no more than 0.15% and ropivacaine no more than 0.2%, in combination with low-dose opioids. At such low dosages, the amount of the analgesic bolus itself can be considered a test dose. A 2005 survey of obstetric anesthesia practices in the UK [13] reported that epidural test doses varied widely, with 75% of institutions considered the administration of a proportion of the therapeutic dose as a test dose.

In this study, we used 0.1% ropivacaine and 2 μg/ml fentanyl in 10 ml as the modified test dose. This is part of the initial dose of labor analgesia (usually 15 ~ 20 ml), which does not require to be configured separately and are very convenient. Our previous study has shown that ropivacaine 5 mg in combination with a low-dose opioid is effective and safe for intrathecal injection as an epidural test dose [14]. Ropivacaine 10 mg is larger than the 5 mg dose in our previous study, so the effectiveness of testing intrathecal administration is beyond doubt. In a classic dose-response study of spinal ropivacaine for cesarean section, subarachnoid injection of ropivacaine 10 mg resulted in successful anesthesia in only 8.3% of women. The ED50 and estimated ED95 for spinal ropivacaine were 16.7 and 26.8 mg, respectively [25]. Therefore, even if 10 ml of 0.1% ropivacaine and 2 μg/ml fentanyl are injected into the subarachnoid space by mistake, it will not cause serious consequences. A recent expert review also emphasized that the initial dose of epidural analgesia is safe as long as it does not exceed the dose of spinal anesthesia for cesarean section and is closely observed after administration [7]. The blocking potency of ropivacaine to bupivacaine is 1:1.3–1.5 [26], and bupivacaine to lidocaine is 4:1 [16]. Thus, the potency of ropivacaine to lidocaine is 2.7–3.1:1. Although we tried to match the efficacy of the local anesthetic as closely as possible between the two test doses, it was clear that 45 mg of lidocaine was more potent in blocking than 10 mg of ropivacaine and approximately equivalent to 15 mg of ropivacaine. This is also evidence that the safety of 10 ml of 0.1% ropivacaine and 2 μg/ml fentanyl as test dose is not inferior to that of traditional test dose. On the other hand, the onset time of lidocaine (5–15 min) is shorter than that of ropivacaine (15–20 min) in epidural anesthesia [16]. Therefore, it seems that replacing traditional test doses with part of an initial analgesic dose may delay the onset of labor analgesia. However, there was no significant difference in the onset time of analgesia between the two regimens in this study, which indicated that ropivacaine combined with fentanyl act as fast as lidocaine when used as a test dose. This may be due to the greater volume of the test dose in group RF than in group L(10 ml vs 3 ml) and fentanyl in group RF can accelerate the onset of local anesthetics [27].

Ideal labor analgesia should be an effective analgesic method that doesn’t affect uterine contraction, duration of labor and maternal motor function. Recently, the concept of “walkable labor analgesia” has been highlighted. Ropivacaine, one of the most commonly used local anesthetics for epidural labor analgesia, can block sensory nerves at low concentrations while preserving motor nerve activity, which enables the parturient to have “walkable labor analgesia” [28]. However, in clinical practice, it has been found that the induction of analgesia after the traditional epidural test dose may affect the motor function of parturients and reduce their walking ability. In the study of Pratt et al. [24], after the test dose of lidocaine 45 mg epidural injection for 3 minutes, 7% of parturients had detectable motor block. Although there was no parturient developed detectable motor block after epidural injection when the dose of lidocaine was reduced to 30 mg, 13% of maternal still subjectively felt heavy legs, which will affect the willingness to move freely. In this study, we didn’t observe obvious motor block in the parturient of both groups. However, the incidence of foot warming and foot numbness in the lidocaine group was significantly higher than that in the ropivacaine-fentanyl group. The feeling of numbness in the lower limbs also affects the willingness of the maternal to get out of bed and move freely.

There are some limitations to our study. First, in the traditional epidural test dose, lidocaine has several options such as 30, 40, and 45 mg, and the medication regimen for initiating labor analgesia also varies greatly in different medical institutions. We only compare one representative dose of each drug, which does not reflect the entire clinical situation. Second, we had only observed for 30 min since the analgesic initiation. Although the motor nerve block of fentanyl and low concentration of ropivacaine is minor, repeated usage may still lead to motor block [1]. Third, NPRS is a relatively subjective outcome for pain and of obvious personal variance. Forth, some samples used oxytocin during labor, which may affect the pain scale. However, there is no difference in oxytocin exposure between the two groups.

## Conclusion

In summary, this study compared the characteristics of epidural labor analgesia induced by the traditional test dose with the use of a portion of the initial analgesic dose as a test dose. The results demonstrate that 0.1% ropivacaine 10 ml combined with 2 μg/ml fentanyl as a modified test dose did not delay the onset of labor analgesia, and the side effects were slightly reduced compared with 1.5% lidocaine 3 ml.

## Data Availability

The datasets used and/or analysed during the current study available from the corresponding author on reasonable request.
